# Herbal medicine for benign prostatic hyperplasia: A protocol for a systematic review of controlled trials

**DOI:** 10.1097/MD.0000000000014023

**Published:** 2019-01-04

**Authors:** Ji Hwan Kim, Kyung Moo Park, Ju Ah Lee

**Affiliations:** a Department of Sasang Constitutional Medicine; b Department of Korean Medicine Rehabilitation; c Department of Internal Medicine, College of Korean Medicine, Gachon University, Seongnam, Korea.

**Keywords:** benign prostatic hyperplasia, herbal medicine, protocol, systematic review

## Abstract

**Background:**

Herbal medicine has been used to manage benign prostatic hyperplasia (BPH) and the associated lower urinary tract symptoms, but its effects are not yet fully understood. The purpose of this review is to assess the efficacy and safety of herbal medicine as a treatment for BPH.

**Methods and analysis:**

Thirteen databases will be searched for relevant studies from inception to the present date. We will include randomized controlled trials assessing herbal medicine for the treatment of BPH. The methodological qualities, including the risk of bias, will be evaluated using the Cochrane risk of bias assessment tool, while confidence in the cumulative evidence will be evaluated using the Grading of Recommendations Assessment, Development and Evaluation (GRADE) approach.

**Ethics and dissemination:**

Ethical approval is not required, as this study is based on the review of published research. This review will be published in a peer-reviewed journal and disseminated both electronically and in print.

## 1 Introduction

Benign prostatic hyperplasia (BPH) is clinically defined as the non-malignant enlargement of smooth muscle and epithelial cells in the prostate. It is often associated with lower urinary tract symptoms (LUTS), including a weak urinary stream, hesitancy, nocturia, and urgency.^[1,2]^

Studies have consistently reported that the risk of BPH increases with age. Although the reported prevalence of BPH/LUTS varies due to differences in its definition, diagnostic methods, and geographic regions,^[3]^ it affects an estimated 50% to 70% of men over the age of 50 years. As age increases from 70 to 80 years or older, the prevalence of BPH also increases from 80% to 88% to 90%.^[4,5]^

Conventional options to manage BPH symptoms include watchful waiting, behavioral modification, medical therapy, and surgery.^[6]^ However, α-blockers, the most frequently prescribed medication for BPH, may cause negative side effects such as hypotension or dizziness.^[7]^ Elderly patients with multiple comorbid diseases such as cardiovascular disease, diabetes mellitus, hypertension, or renal impairments are at an increased risk for drug-related side effects^[8]^ and the need for anesthesia.^[9]^

Complementary and alternative therapies have also been used to treat BPH. A systematic review of the effect of acupuncture on BPH reported that acupuncture caused statistically significant changes in the short-term follow-up endpoints of patients with moderate to severe BPH.^[10]^ A meta-analysis of clinical trials examining the use of moxibustion suggested that it is also effective in treating BPH patients.^[11]^ In the USA, 40% of men who choose non-surgical treatment use herbal supplements alone or with other medications.^[12]^ A systematic review on the efficacy and safety of Chinese herbal medicine for BPH has been published in 2013.^[13]^ Since the time of the search for the included research in this review is 2011, it is considered necessary to update the review.

Therefore, the purpose of this study is to systematically review randomized controlled trials (RCTs) from inception to 2018 in order to assess the efficacy and safety of herbal medicine for the treatment of BPH.

## 2 Methods

### 2.1 Study registration

The current protocol report adheres to the Preferred Reporting Items for Systematic Reviews and Meta-Analysis (PRISMA) Protocols.^[14]^ The protocol for this systematic review has been registered in PROSPERO 2018 under number CRD42018087807.

### 2.2 Eligibility criteria

#### 2.2.1 Types of studies

Prospective RCTs and quasi-RCTs evaluating the efficacy of herbal medicine for the treatment of BPH will be included in this review. Both treatments with herbal medicine alone and concurrent treatment using herbal medicine and another therapy will be considered acceptable if herbal medicine is administered to the intervention group only and any other treatment is administered equally to both groups. Trials with any type of control intervention will be included. However, we will exclude studies comparing different types of herbal formulas. There will be no restrictions on publication language.

#### 2.2.2 Types of participants

This study will include patients with symptomatic BPH. Participants with diagnoses of other diseases that may cause LUTS will be excluded.

#### 2.2.3 Types of interventions

All types of herbal medicine for BPH (i.e., decoction, tablet, pill, powder, capsule, extract) will be considered for inclusion. This review will only include studies that used herbal medicines prescribed by traditional East Asian medicine doctors.

### 2.3 Types of outcome measurements

#### 2.3.1 Primary outcomes

Primary outcome measures will include changes in urological symptoms as measured by validated urologic symptom scores such as the International Prostate Symptom Score, the American Urologic Association Symptom Score,^[15]^ and the Boyarsky Score.^[16]^

#### 2.3.2 Secondary outcomes

Secondary outcomes will include the Quality of Life score, changes in prostate size (cc), and various urodynamic measures such as the number of voids at night (nocturia), residual urine volume (mL), mean urine flow (mL/sec), and peak urine flow (mL/sec). The number and severity of adverse events will also be recorded.

### 2.4 Data sources and search strategy

Thirteen electronic databases will be searched from inception to the present date:

1) Cochrane Central Register of Controlled Trials (CENTRAL)
2) MEDLINE
3) EMBASE
4) Oriental medicine Advanced Searching Integrated System (OASIS)
5) The Korean Traditional Knowledge Portal
6) The Korean Studies Information Service System
7) KoreaMed
8) The Korean Medical Database
9) DBPIA
10) China National Knowledge Infrastructure (CNKI)
11) Wanfang
12) VIP
13) J-STAGE.

The MEDLINE database search strategy is presented in Supplement 1. Similar search strategies will be used for the other databases.

### 2.5 Data extraction, management, and assessment of risk of bias

Hard copies of all articles will be obtained and read in full. Two authors (JHK and JYC) will extract the data and assess the quality using a predefined data-extraction form. The risk of bias will be evaluated using the Cochrane risk of bias assessment tool, version 5.1.0, which takes into account random sequence generation, allocation concealment, blinding of participants and personnel, blinding of the outcome assessment, completeness of outcome data, selective reporting, and other sources of bias.^[16]^ The results of these evaluations will be presented using the scores “L”, “U”, and “H” to indicate a low risk of bias, an uncertain risk of bias, and a high risk of bias, respectively. Disagreements will be resolved by discussion among all authors. When disagreements regarding selection cannot be resolved through discussion, an arbiter (JAL) will make the final decision.

### 2.6 Data analysis

#### 2.6.1 Data synthesis

Differences between the intervention and control groups will be assessed. The mean difference (MD) and a 95% confidence interval (CI) will be used to measure the effects of treatment for continuous data. We will convert other forms of data into the MD. For outcome variables on different scales, we will use the standard MD and a 95% CI. For dichotomous data, we will present treatment effects as the relative risk and a 95% CI; other binary data will be converted into the relative risk.

All statistical analyses will be conducted using the Cochrane Collaboration's software programme Review Manager version 5.3 for Windows (Copenhagen, The Nordic Cochrane Centre, the Cochrane Collaboration, 2012). We will contact the corresponding authors of studies with missing information to acquire and verify data whenever possible. As appropriate, we will pool data across studies to conduct a meta-analysis using fixed- or random-effects models. We will use Grading of Recommendations Assessment, Development and Evaluation (GRADE) pro software from Cochrane Systematic Reviews to create a Summary of Findings table.

#### 2.6.2 Unit of analysis issues

For crossover trials, data from the first treatment period will be used. For trials assessing more than 1 control group, the primary analysis will combine data from each control group. Subgroup analyses of the control groups will be performed. Each patient will be counted only once in the analyses.

#### 2.6.3 Dealing with missing data

Intention-to-treat analyses, including all randomized patients, will be performed. For patients with missing outcome data, a last observation carry-forward analysis will be performed. When individual patient data are initially unavailable, we will review the original source or the published trial reports.

#### 2.6.4 Assessment of heterogeneity and subgroup analysis

Based on our data analyses, we will use random- or fixed-effects models to conduct the meta-analysis. Chi-squared and I^2^ tests will be used to evaluate the heterogeneity of the included studies, with I^2^ > 50% indicating high heterogeneity. When heterogeneity is observed, subgroup analyses will be conducted to explore the possible causes.^[17]^

#### 2.6.5 Assessment of reporting biases

Funnel plots will be generated to detect reporting biases when a sufficient number of included studies (at least 10 trials) are available.^[18]^ However, because funnel plot asymmetry is not equivalent to publication bias, we will aim to identify the possible reasons for any asymmetry in the included studies such as small-study effects, poor methodological quality, or true heterogeneity.^[19,20]^

### 2.7 Ethics and dissemination

Ethical approval is not required, as this study is based on a review of published literature. The results of this review will be disseminated electronically and in print through a peer-reviewed publication.

## 3 Discussion

The purpose of this review is to assess the efficacy and safety of herbal medicine as a treatment for BPH. Systematic reviews examining the efficacy of both acupuncture^[10]^ and moxibustion^[11]^ for BPH have already been published. In addition to acupuncture, herbal medicine is frequently used in Asia for the treatment of BPH, but its effects are not yet fully understood. This review will provide evidence on the efficacy and safety of herbal medicine for the treatment BPH, which will be helpful for patients, practitioners, and healthcare providers.

## Author contributions

**Conceptualization:** Ji Hwan Kim.

**Data curation:** Ji Hwan Kim, Kyung Moo Park.

**Formal analysis:** Ji Hwan Kim, Kyung Moo Park.

**Funding acquisition:** Ju Ah Lee.

**Project administration:** Ju Ah Lee.

**Writing - original draft:** Ji Hwan Kim.

**Writing – review & editing:** Kyung Moo Park, Ju Ah Lee.

Ji Hwan Kim orcid: 0000-0001-7270-0987.
